# Terpene content in the air of younger and older forests in southeastern Poland: implications for forest therapy

**DOI:** 10.1038/s41598-025-26137-3

**Published:** 2025-11-26

**Authors:** Tomasz Dudek, Mariusz Marć

**Affiliations:** 1 Department of Agroecology and Forest Utilization, Faculty of Technology and Life Sciences, University of Rzeszów, Ćwiklińskiej Str. 1, 35-601 Rzeszow, PL Poland; 2 Department of Analytical Chemistry, Faculty of Chemistry, Gdańsk University of Technology, Narutowicza Str. 11/12, 80-233 Gdańsk, PL Poland; 3 Departamento de Medio Ambiente, INIA, Carretera de A Coruña Km 7, 28040 Madrid, Spain

**Keywords:** Air quality, Monoterpenes, TVOC, Forest bathing, Forest therapy, Ecology, Ecology, Environmental sciences, Plant sciences

## Abstract

The aim of the study was to investigate differences in terpene content level in the air within younger and older forests of the same species. Conducted from July 18 to October 2 in Nemoral Scots pine and Central European submontane beech forests, air samples were collected using portable aspirators with Tenax TA sorbent tubes. The compounds were identified and quantified using gas chromatography with flame ionization detection. Results showed that older forests emitted more volatile organic compounds (VOCs) than younger ones. Regardless of forest age or species, α-pinene was the most prevalent terpene, accounting for 7.4–12.9% of total VOCs. Other terpenes, such as β-pinene and camphene, were found in lower concentrations (1.7–12.7 times less). In Scots pine, higher α-pinene concentrations were observed in older forests, while younger forests had higher levels of 3-carene and α-terpinene. For beech, higher concentrations of camphene and β-pinene were found in younger forests. In nearly half of the cases, TVOC values and terpene concentrations showed moderate variability (25–45%), with high variability (> 45%) recorded in the remainder. Considering the quantitative and qualitative structure of terpenes, an older pine forest would be most suitable for supporting the treatment of respiratory diseases.

**Supplementary Information:**

The online version contains supplementary material available at 10.1038/s41598-025-26137-3.

## Introduction

Science provides evidence for the positive effects of terpenes and terpenoids on mental health^1–3^, physical health^4–8^, and immune function, including cancer resistance^9–11^, which is important for health prevention. Terpenes are produced by plants, and forests are the main emitters of these compounds into the atmosphere^12–14^. It is worth noting that in addition to living trees, leaf litter is another important source of terpenes in the atmosphere, releasing them during decomposition at a rate comparable to viable needles^15^. Many studies have reported that terpenes are likely the main contributors to the positive health effects of such activities in forest environment as forest recreation and forest bathing^16–18^.

Most of the works devoted to organic compounds produced/emitted by trees into the atmosphere present results without describing the species composition of the forest stands (e.g^19–21^). Additionally, some studies measured terpene content in leaves^22–25^ or above the tree canopy^26,27^, rather than in the air within the forest interior, which is what forest visitors breathe. The terpene content level in forest air may determine the applicability of forest therapy in a particular type of forest. Research on terpene composition in forest air has been conducted with respect to *Abies* sp.^28,29^, *Larix cajanderi*^30^, *Picea abies*^31^, *Pinus* sp.^32^, Aaltonen et al., 2010;^33,34^, *Fagus silvatica*^33^, and *Quercus ilex*^35^. However, none of these studies compared terpene emissions in forests of the same species but different age classes. Plant physiology studies indicate that the qualitative structure of terpenes and the emission rate depend not only on the species^33,36^ but also on the age of the plant^37^.

The purpose of this study was to investigate potential differences in terpene levels in the air within younger and older forests of the same species. The study aimed to determine whether such differences could be observed in a coniferous forest (using *Pinus sylvestris* L.) and a deciduous forest (*Fagus sylvatica* L.). Therefore, we determined the quantitative and qualitative structure of VOCs in younger and older Scots pine forests (49 and 78 years old) and beech forests (42 and 92 years old).

## Materials and methods

### Description of sampling area and location of sampling points

**Fig. 1:**
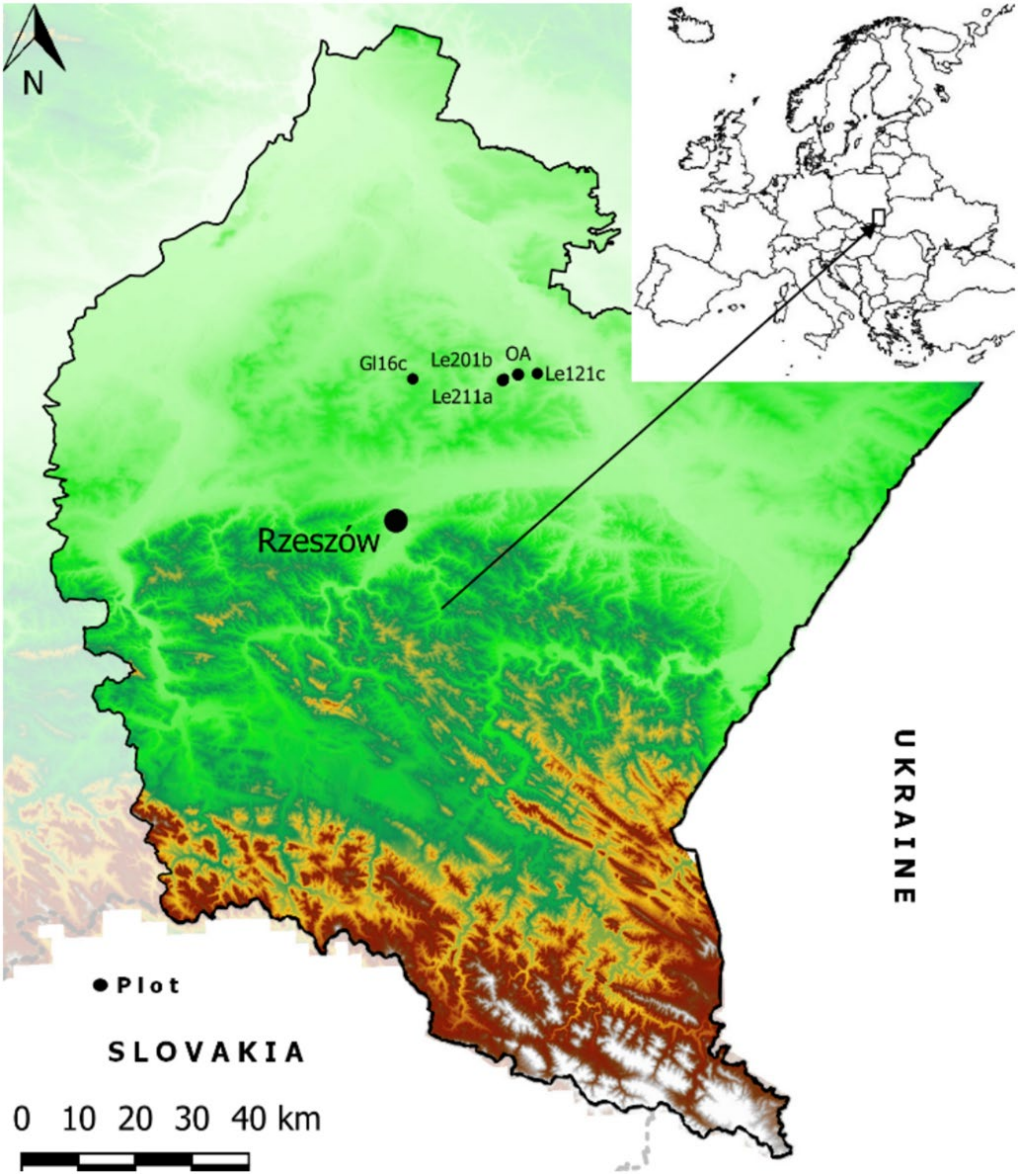
The general view of the study area, southeaster Poland, in the Głogów (Gl) and Leżajsk (Le) Forest Districts.

The study was conducted from July 18 to October 2, 2024, and included four measurement campaigns. The research areas were located in two of the most common forest types in the temperate climate zone of Europe: Nemoral Scots pine forest and Central European submontane beech forest in southeastern Poland, in the Głogów (Gl) and Leżajsk (Le) Forest Districts (Fig. 1).

The forest cover of this region is one of the highest in Poland, reaching 38.3%, compared to the national average of 29.7%^38^. Additionally, a control area was established in an open agricultural landscape, approximately 200 m from the forest edge.

The studies in both forest types were conducted on the same days, and the stands were located in the same region. Therefore, meteorological factors that can influence VOC content, such as air temperature and humidity, wind speed and direction, were comparable across sites and did not affect the relative differences in VOC concentrations between the compared forest types.

The climate of the region is characterized by the following indicators, averaged over the years 2019–2023: (1) average annual temperature: 10.0 °C; (2) total annual precipitation (rain or snow): 667 mm; (3) annual average humidity: 74%; (4) average length of the growing season: 225–230 days; (5) prevailing wind direction: S-W, W, N-W^39^, www.tutiempo.net). The Głogów Forest District area is flat, slightly undulating, with minor terrain denivelations. The absolute elevations range from 197 to 240 m above sea level. The dominant soil types in the area are podzolic and rusty soils. The forests are dominated by Scots pine (77.2%), oak species (6.4%), alder species (5.3%), and common beech (3.0%)^40^. The area of the Leżajsk Forest District is more diverse, with an elevation difference of 89 m (165–254 m above sea level). The dominant soil types in this area are rusty and podzolic soils. The forests are dominated by Scots pine (70.3%), common beech (9.4%), oak species (6.0%), and silver fir (5.3%)^41^. The terrain was flat across all sampling areas.

The following criteria were used when selecting forest stands for the study: two pine stands with at least 90% pine content and two beech stands with at least 90% beech content; the age of the younger stands was at least 30 years (younger stands, under 30 years old, are very dense, and additionally, in Poland, there is a year-round entry ban to forests with a height of up to 4 m) and no more than 50 years; the age of the older stands was at least 70 years. Four forest stands were selected using the Forest Data Bank (BDL; https://www.bdl.lasy.gov.pl/portal), with their locations and characteristics listed in Table 1. Additionally, a control area was established near the selected forest complexes in an open agricultural landscape, approximately 200 m from the forest edge.

**Table 1:** Characteristics of stand parameters based on the forest management plan (2021a, b) and location of research plots.

Forest district units	Coordinates (°)	Area (ha)	SC (%)	Admixture species	Age (Years)	UU (%)	H (m)
Gl16c	N50.2464 E22.0568	4.67	100P	Q, F	78	30	21
Le121c	N50.2605 E22.3650	9.93	90P; 10Pi	Q, F, C, B, L	49	30	23
Le201b	N50.2553 E22.2830	6.96	100F	Q, B, C, P	92	40	29
Le211a	N50.2523 E22.2789	15.32	50F; 40A; 10Pi*	C, B, L, P	42	30	23

Gl: Głogów Forest District; Le: Leżajsk Forest District; SC: tree species composition; P: *Pinus sylvestris*; Q: *Quercus robur*; F: *Fagus sylvatica*; C: *Carpinus betulus*; A: *Abies alba*; B: *Betula pendula*; Pi: *Picea abies*; L: *Larix decidua*; UU: undergrowth and underbrush; H: height of tree stands according to the dominant species. * In the sampling area, the forest composition was 90% Fagus sylvatica and 10% Abies alba.

### Sampling strategy and organic compound determination conditions

Sampling of analytes present in atmospheric air in the forest area was carried out dynamically using aspirators (individual aspirator type AP-8ch, S.I. “TWO-MET”, Zgierz, Poland) connected to tubes containing a sorption bed. Isolation and enrichment of analytes from the gaseous phase were carried out using steel tubes filled with polymer-based sorbent Tenax TA (O.D. × I.D. × L: 1/4 in. × 3 1/2 in., preconditioned, 60–80 mesh; Merck KGaA, Darmstadt, Germany). The tubes were conditioned at 300 °C for 30 min prior to each sampling period according to the manufacturer’s guidelines.

The aspirators combined with sorption tubes were installed at 9 sampling points in each of the forest areas studied. Sampling of analytes was conducted for 150 min at a constant flow rate of 350 mL min^-1^. Samples were collected from at a height of 1.5–1.8 m above the ground. Aspirators were mounted on tripods rather than on trees or branches. After the sampling period, tubes were secured with brass nuts with Teflon seals and transported to the laboratory for further analysis. Release, separation and determination of the analytes were performed within three days of the sampling campaign.

The release, separation and final determination of organic compounds retained on the Tenax TA sorption medium was carried out following an analytical protocol described in detail elsewhere^42–44^. Briefly, the release of analytes from the sorption medium was performed using a solventless extraction technique—two-stage thermal desorption (TD, Markes Series 2 Thermal Desorption Systems; UNITY/TD-100, Markes International, Inc.) – under the following conditions: extraction temperature – 285° C, extraction time – 12 min, microtrap temperature during first desorption stage – 0° C, microtrap temperature during second desorption stage – 300° C (by 5 min), inert gas flow rate during first desorption stage – 50 mL/min, and inert gas flow rate during second extraction stage – 2.0 mL min^-1^. The separation, identification and final determination of organic compounds released from sorption medium were conducted using a gas chromatography-flame ionization detector (GC-FID) system (Agilent Technologies 7820A GC System) under the following conditions: transfer line temperature (TD-GC connection) – 180° C; FID working temperature – 280° C, GC capillary column – (30 m × 320 μm × 5 µm; J&W DB-1, USA); helium flow rate – 2.0 mL·min^-1^, and oven parameters initially set at 45 °C for 1 min, then increased at a rate of 15° C min^-1^ to 120° C and held for 1 min, then increased at a rate of 7° C min^-1^ to 260° C and maintained for 6 min. Operating software was OpenLab CDS (Agilent Technologies, Inc.)

### Qualitative and quantitative analysis of terpenes representatives

A commercially available reference standard solution (Cannabis Terpene Mix A certified reference material, TraceCERT®, Merck KGaA, Darmstadt, Germany) was used for qualitative analysis of terpene representatives. This solution contained 2000 μg mL^-1^ of representative terpenes, including β-pinene; camphene; α-pinene; 3-carene; α-terpinene and (R)-( +)-limonene in methanol. The identification of specific chemical compounds was performed by comparing the retention times from the reference solution analysis with the chromatograms obtained after analyzing the actual samples (analytes adsorbed on the sorption medium). The identification error margin was set at 2.5%. The Kovats retention indices (LRI) of selected terpenes representatives have been estimated and presented in a previous paper^33^. Examples of chromatograms obtained during the analysis of samples collected from the air over forest and open areas are enclosed in the supplementary materials (Supplementary Figs. 1, 2, 3).

A five-point calibration curve was prepared for the quantitative analysis of the mass of analytes retained on the sorption medium during sampling. Calibration solutions were prepared in a concentration range from 4 to 1000 µg mL^-1^. Using a glass syringe, 1 µL of each calibration solution was applied to a clean Tenax TA sorption bed, with each concentration repeated three times. The tubes containing defined terpene concentrations were then gently flushed with inert gas (nitrogen) for 5 min at a flow rate of 25 mL min^-1^ to evenly distribute the compounds in the sorption medium. Subsequently, the tubes containing reference solutions were analyzed under the same TD-GC-FID conditions as the samples collected from the forest areas. The correlation coefficients (R^2^) of the calibration curves ranged from 0.987 to 0.997. Detailed information about the equipment and working parameters used during the calibration procedure is available in previous studies^42–44^.

For the evaluation of screening parameter defined as TVOC (total volatile organic compound concentration) in the forest air under study, TVOC values were calculated following a literature-based literature protocol. TVOC is typically defined as the sum of all organic compounds eluting between n-hexane and n-hexadecane (defined as the analytical window) on non-polar/slightly polar GC capillary column stationary phases. The analysis used an FID detector and quantified compounds as toluene equivalents^45,46^. For this purpose, a certified reference solution of toluene (200 μg mL^-1^ in methanol) was applied (TraceCERT®, Merck KGaA, Darmstadt, Germany).

To ensure the quality of the results, a blank analysis was performed before each measurement series to check for potential signals from the system or the pure sorption bed. The efficiency of the thermal desorption process (extraction efficiency) was then verified using the previously described standard solutions. The values of extraction efficiency oscillated between 95 and 97%. The limit of detection (LOD) and limit of quantitation (LOQ) were estimated taking into account the signal-to-noise ratio (S/N ratio). The average LOD value was 0.5 ng per sorption tube and the LOQ was 1.5 ng per sorption tube.

### Statistical analysis

The following null hypothesis was tested: there are no differences between the average concentrations of the terpenes studied in the air of younger and older forests, separately for pine and beech stands. Where preliminary analyses confirmed the normal distribution of organic compounds (only in pine forests: α-pinene, 3-carene, and α-terpinene), the t-test for independent samples was used for analysis. However, in most cases, preliminary analyses showed a lack of normal distribution (for all compounds tested in beech forests and for camphene, β-pinene in pine forests), prompting the use of the Mann–Whitney U test for further statistical analysis. The level of statistical significance was set at P < 0.05. All analyses were performed using Statistica 13 software.

## Results

The values of the total TVOC parameter indicated that older stands emitted significantly higher amounts of VOCs into the atmosphere compared to younger ones. This was particularly evident in the Scots pine forest (Table 2), where the differences were statistically significant (Z = 3.1466, p = 0.0016). The variability in the obtained results may be partly attributed to the fact that only a defined volume of air is passed through the sorption medium – not entire cubic meter. Moreover, the investigated medium – air over the forest area – is a non-homogeneous medium. For this reason, it is another factor that might affect the obtained research results, contributing to the higher fluctuation in the data. In addition, potential variability may also arise from the sorbent material itself, since slight inhomogeneities in the Tenax TA packing can influence adsorption efficiency for specific compounds – minor differences in a mass of sorbent, its surface area or particles shape. Furthermore, operational non-uniformities of the applied aspirators, such as fluctuations in flow stability, may be an additional factor which slightly contribute to variability and uncertainty in the measured concentrations.

**Table 2:** Results of descriptive statistics for the studied compounds (µg m^-3^) and locations.

Variable	α-pinene	Camphene	β-pinene	3-carene + α-terpinene	D-limonene	TVOC
Le121c—younger pine forest						
min/max	0.348/1.703	0.084/0.317	0.053/0.219	0.024/0.201	< LOD	2.582/17.310
Average	0.941	0.169	0.133	0.102	< LOD	7.286
SD	0.444	0.070	0.046	0.045	0.000	3.713
CI95	0.643/1.239	0.116/0.222	0.104/0.162	0.064/0.140	< LOD	4.927/9.645
Gl16c—older pine forest						
min/max	0.572/2.524	0.061/0.351	0.059/0.275	0.019/0.107	0.067/0.198	6.047/34.587
Average	1.558	0.154	0.136	0.047	0.111	18.516
SD	0.634	0.116	0.073	0.034	0.061	9.109
CI95	1.132/1.984	0.057/0.251	0.09/0.182	0.011/0.083	0.013/0.209	12.728/24.30
Le211a—younger beech forest						
min/max	0.594/2.181	0.073/2.325	0.091/0.686	0.077/0.261	0.066/0.741	8.626/32.224
Average	1.329	0.856	0.271	0.125	0.220	17.944
SD	0.518	0.683	0.188	0.061	0.201	6.746
CI95	1.000/1.658	0.397/1.315	0.144/0.398	0.074/0.176	0.052/0.388	13.658/22.23
Le201b—older beech forest						
min/max	0.573/2.989	0.067/0.165	0.053/0.210	0.058/0.145	0.058/0.103	10.294/36.56
Average	1.544	0.106	0.121	0.091	0.085	18.364
SD	0.889	0.034	0.046	0.026	0.017	8.170
CI95	0.979/2.109	0.07/0.142	0.092/0.150	0.071/0.111	0.067/0.103	13.173/23.55
Open area						
min/max	0.384/1.317	0.065/0.118	0.078/0.315	< LOD	< LOD	5.195/24.326
Average	0.791	0.084	0.155	< LOD	< LOD	12.830
SD	0.310	0.022	0.086	< LOD	< LOD	5.534
CI95	0.553/1.029	0.057/0.111	0.089/0.221	< LOD	< LOD	8.576/17.084

An interesting observation was that the VOCs concentration in the open area, located about 200 m from the forest edge, was 57% higher than inside the younger Scots pine forest. The Mann–Whitney U test also revealed statistically significant differences in this case (Z = 2.4518, p = 0.0142). In almost half of the cases, the total VOCs concentration in the air, as well as the levels of individual terpenes, showed moderate variability, with the mean value effectively reflecting the concentration of the organic compounds studied in the forest air. For nearly half of the cases, the coefficient indicated a high level of variability, making it more appropriate to use the range (min – max) rather than the average. In three cases, the coefficient of variation could not be calculated due to the low concentration of compounds (< LOD) in the air in the research areas, while in one case, low variability of compounds present in the air was observed (Table 3).

**Table 3:** Coefficient of variation V [%] for the TVOC parameter and representatives of the terpene group identified in the air at the experimental sites.

	Open area	Gl16c	Le121c	Le211a	Le201b
TVOC	43 ↑↑	49 ↑↑↑	51 ↑↑↑	38 ↑↑	44 ↑↑
α-pinene	39 ↑↑	41 ↑↑	47 ↑↑↑	37 ↑↑	58 ↑↑↑
camphene	26 ↑↑	75 ↑↑↑	41 ↑↑	80 ↑↑↑	32 ↑↑
β-pinene	58 ↑↑↑	223 ↑↑↑↑	35 ↑↑	65 ↑↑↑	38 ↑↑
3-carene and α-terpinene	–	73 ↑↑↑	40 ↑↑	49 ↑↑↑	28 ↑↑
D-limonene	–	55 ↑↑↑	-	91 ↑↑↑	20 ↑

↑ – V < 25% low variability; ↑↑ – 25% ≤ V < 45% moderate variability; ↑↑↑ – 45% ≤ V ≤ 100% high variability; ↑↑↑↑ – V > 100% very high variability; TVOC – total volatile organic compounds.

The highest average concentration of α-pinene was recorded in the air of the older Scots pine forest (1.558 µg m^-3^) with a variability coefficient (V) of 41%, while the lowest was observed in the open area near the forest (0.791 µg m^-3^) at V = 39%. The highest maximum value was recorded in the older beech forest (2.989 µg m^-3^), while the lowest was observed in the younger Scots pine forest (0.348 µg m^-3^) (Tables 2, 3).

The highest average concentration of camphene was recorded in the younger beech forest (0.856 µg m^-3^) at V = 80%. It should be noted that fir trees were present in this area near one of the air sampling points within the stand, and the high coefficient of variation requires caution when interpreting the average value. The highest average concentration of camphene was recorded in the younger beech forest (0.856 µg m^-3^) at V = 26%. The highest maximum concentration was observed in the younger beech forest (2.325 µg m^-3^), while the lowest was recorded in the air within the younger pine forest (0.061 µg m^-3^). However, the minimum values across all sites were very similar (Tables 2, 3).

The highest average concentration of β-pinene was recorded in the air sampled from the younger beech forest (0.271 µg m^-3^) at V = 65%, while the lowest was in the air within the older beech forest (0.121 µg m^-3^) at V = 38%. The highest maximum concentration of this compound in the air was recorded in the younger beech forest (0.686 µg m^-3^), while the lowest, identical value was observed in both the younger pine forest and the older beech forest (0.053 µg m^-3^) (Tables 2, 3).

The highest maximum concentration of this compound in the air was recorded in the younger beech forest (0.686 µg m^-3^), while the lowest, identical value was observed in both the younger pine forest and the older beech forest (0.053 µg m^-3^) (Table 2). The highest maximum concentration was recorded in the younger beech forest (0.261 µg m^-3^), while the lowest was observed in the older pine forest (0.107 µg m^-3^) (Table 2).

The highest average concentration of D-limonene in the air on the monitored areas was recorded in the younger beech forest (0.220 µg m^-3^) at V = 91%, while the lowest was found in the open space air and young pine forest (< LOD). The highest maximum concentration in the air was recorded in the younger beech forest (0.741 µg m^-3^), while the lowest was observed in the air over the older beech forest area (0.058 µg m^-3^) (Tables 2, 3).

Interpretation of the t-test results indicates the presence of statistically significant differences in the levels of α-pinene, 3-carene, and α-terpinene in the air depending on the age of the pine forest (Table 4).

**Table 4:** Results of the t-test: group 1 GL16c, group 2 Le121c.

Variable	Average GL16c	Average Le121c	t	df	P
α-pinene	1.5584	0.9409	2.6038	20	0.0170
3-carene and α-terpinene	0.0465	0.1120	− 2.8281	12	0.0152

Higher concentrations of α-pinene were observed in the air over the older pine forest area, while 3-carene and α-terpinene were more prevalent in the air over the young pine forest area (Table 2).

The data obtained after calculating the Mann–Whitney U test revealed significant differences in the levels of camphene and β-pinene in the air of younger and older beech forests (Table 5).

**Table 5:** Results of the Mann–Whitney U test.

Variable	Sum of range		Z	p
Nemoral Scots pine forest				
	Le121c	GL16c		
β − pinene	155	145	− 0.2598	0.7950
Camphene	93	78	− 0.6181	0.5365
Central European submountainous beech forests				
	Le211a	Le201b		
α − pinene	147	153	− 0.1443	0.8852
β − pinene	183	93	3.1081	0.0019
Camphene	127	26	2.7638	0.0057
3 − carene and α − terpinene	83	70	1.0103	0.3123
D − limonene	74	31	1.7428	0.0814

In both cases, significantly higher concentrations of these compounds were observed in the air of the younger beech forest (Table 2). For the remaining terpenes, noticeable differences could be seen in the concentration of D-limonene in the air of beech forests of different age classes (Table 2), but they were not statistically significant.

## Discussion

In almost half of the cases, the total VOCs concentration (defined as the numerical value of the TVOC parameter), and the concentrations of individual terpenes detected in the air showed moderate variability (25–45%), while the remaining cases had high variability (> 45%). This represents a novel finding, as previous research only allowed for the conclusion that the amount of terpenes released into the atmosphere is highly variable^20,33,47^. Such variability is largely influenced by factors such as seasonal changes and associated meteorological conditions^6,48–51^ , soil properties^52^, and stressors affecting trees, such as drought or pathogen attacks ^19^. In addition rainfall in the days preceding the measurements or local wind conditions within the forest stands—could potentially influence absolute VOC levels^53,54^. The average of total VOCs concentration in atmospheric air, defined by the TVOC parameter, was 7.286 µg m^-3^ in the 49-year-old pine forest and more than twice as high in the 78-year-old pine forest – 18.516 µg m^-3^. Dudek et al.^33^ obtained more than two times lower numerical values of the TVOCs parameter for a 78-year-old pine stand (8.394 µg m^-3^) and a similar one for a 77-year-old stand (7.955 µg m^-3^). The highest proportion of terpenes in the air of pine forests of both age classes was recorded for α-pinene: 1.5584 µg m^-3^ (8.4% TVOC) in the older forest and 0.9409 µg m^-3^ (12.9% TVOC) in the younger one. The interpretation of the results confirms previous reports that α-pinene is abundantly produced (and emitted into the atmosphere) by coniferous trees of the family Pinaceae (Tisseran et al., 2013). Aaltonen et al.^55^ also identified α-pinene (with an emission rate of 2.975 µg m^-2^ h^-1^) as the most prevalent compound in the air of a pine forest in southern Finland. Staudt et al.^34^, in their study on pine forests in southwestern France, also found that α-pinene and β-pinene represented the primary components (80–90%) of VOC emissions. Given the potential application of forest air quality data in forest therapy, it is appropriate to present the results in µg m^-3^. This unit allows for easy calculation of the inhalation time in forest air to deliver an appropriate dose of specific terpenes to the body. The results of a study conducted by Sumitomo et al.^56^ have demonstrated that monoterpenes, including α-pinene, easily penetrate from forest air through the respiratory tract into the body and accumulate in the serum. Inhaling forest air rich in monoterpenes (50% α-pinene or 16% sabinene) could have improved the effectiveness of asthma treatment in adolescents^57^. Inhaling forest air rich in limonene and pinene may exert anti-inflammatory effects on the respiratory system and improve well-being, cognitive function, and psychomotor condition^16^. Additionally, pinene and linalool may offer new therapeutic solutions in preventing or treating conditions such as stroke, ischemia, inflammatory pain, migraines, cognitive impairment (important for Alzheimer’s disease and aging), insomnia, anxiety, and depression^3^.

Previous studies indicate that α-pinene is also the most frequently detected terpene in the air of beech forests. The average concentration of α-pinene in the air of a 42-year-old beech forest was 1.329 µg m^-3^, accounting for 7.4% of the TVOC value (17.944 µg m^-3^). The concentration of α-pinene in the air of a 92-year-old forest was 1.544 µg m^-3^, accounting for approximately 8.4% of the total VOCs concentration (TVOC = 18.364 µg m^-3^). As for the results published by Dudek et al.^33^, the total VOCs concentration in the atmospheric air of a 76-year-old beech forest was 25.005 µg m^-3^, with α-pinene being the most abundant terpene, accounting for 1.3% (0.329 µg m^-3^). On the other hand, for the 79-year-old forest, where the TVOC value was 11.361 µg m^-3^, α-pinene accounted for 3.0% of VOC (0.337 µg m^-3^).

The other terpenes detected in the forest air had significantly lower concentrations than α-pinene. The second most abundant compound in the air of the older pine forest was β-pinene, with a concentration of 0.3455 µg m^-3^ (1.9% TVOC). In the air of the younger pine forest, the average concentration of camphene was 0.1685 µg m^-3^, which accounted for 2.3% of the total VOC concentration. A similar phenomenon and characteristics were observed in the beech forest areas. The air of the older one had a higher concentration of β-pinene, with 0.1214 µg m^-3^ (0.7% of TVOCs), while the younger forest it was camphene – 0.8564 µg m^-3^ (4.8% of TVOC). The contribution of other terpenes to the total VOCs concentration in the atmospheric air of the area under study was very low, ranging from 0.5 to 1.5% of TVOC for individual compounds.

According to the literature, camphene has the potential to lower cholesterol and triglyceride levels^58^, protect the heart from ischemia/reperfusion damage, and offer therapeutic benefits in mitigating the adverse effects of heart ischemia/reperfusion^59^. The compound may also potentially alleviate skeletal muscle atrophy by regulating oxidative stress and lipid metabolism^60^. It also exhibits anticancer properties in melanoma^4^ and a broad spectrum of analgesic effects^61^.

Three out of the four study areas located within the forest stands contained higher concentrations of TVOCs compared to the open land adjacent to the forest. The higher TVOC value observed in the air of the open area bordering the forest, compared to the air inside the 49-year-old pine forest, could result from the high tree density in the younger pine forest, which restricts air exchange between the canopy and the forest interior. A similar situation applied to the presence of tree pollen, which was more abundant in the air of the areas bordering the forest than inside the forest, beneath the tree canopies^62^.

## Conclusions

- Our results indicate that older forests produce more TVOC than younger ones, with this difference being particularly noticeable in pine forests, while smaller variations were observed in beech forests.
- Regardless of forest age or species, α-pinene had the highest concentration in the air of the forest interiors. Its content varied from 7.4 to 12.9% of TVOC. The concentration of other terpenes (β-pinene and camphene) was lower, on average, by a factor of 1.7 to 12.7.
- Significant differences were found in α-pinene, 3-carene and α-terpinene content depending on the age of the pine forest. Higher amounts of α-pinene were found in the older pine forest, while 3-carene and α-terpinene were more abundant in the younger forest.
- Significant differences in the content of camphene and β-pinene were observed based on the age of the beech forest. In both cases, significantly higher concentrations of these compounds were recorded in the air of the younger beech forest.
- In nearly half of the cases, the content of TVOC and individual terpenes in the air showed moderate variability (25–45%), while the other half exhibited high variability (> 45%).
- Considering the quantitative and qualitative structure of terpenes, an older pine forest would be most suitable for supporting the treatment of respiratory diseases, while a younger beech forest would be better suited for therapies related to cardiovascular conditions.

## Supplementary Information

Supplementary Information.

## Data Availability

The datasets used and/or analysed during the current study available from the corresponding author on reasonable request.
