# Supplementary material for: Terpene content in the air of younger and older forests in southeastern Poland: implications for forest therapy
Source: Sci Rep. 2025 Nov 26;15:42174. doi: 10.1038/s41598-025-26137-3 (PMC12657507; doi:10.1038/s41598-025-26137-3)
Supplement: Supplementary file 1 — Supplementary Information. [file 41598_2025_26137_MOESM1_ESM.docx]

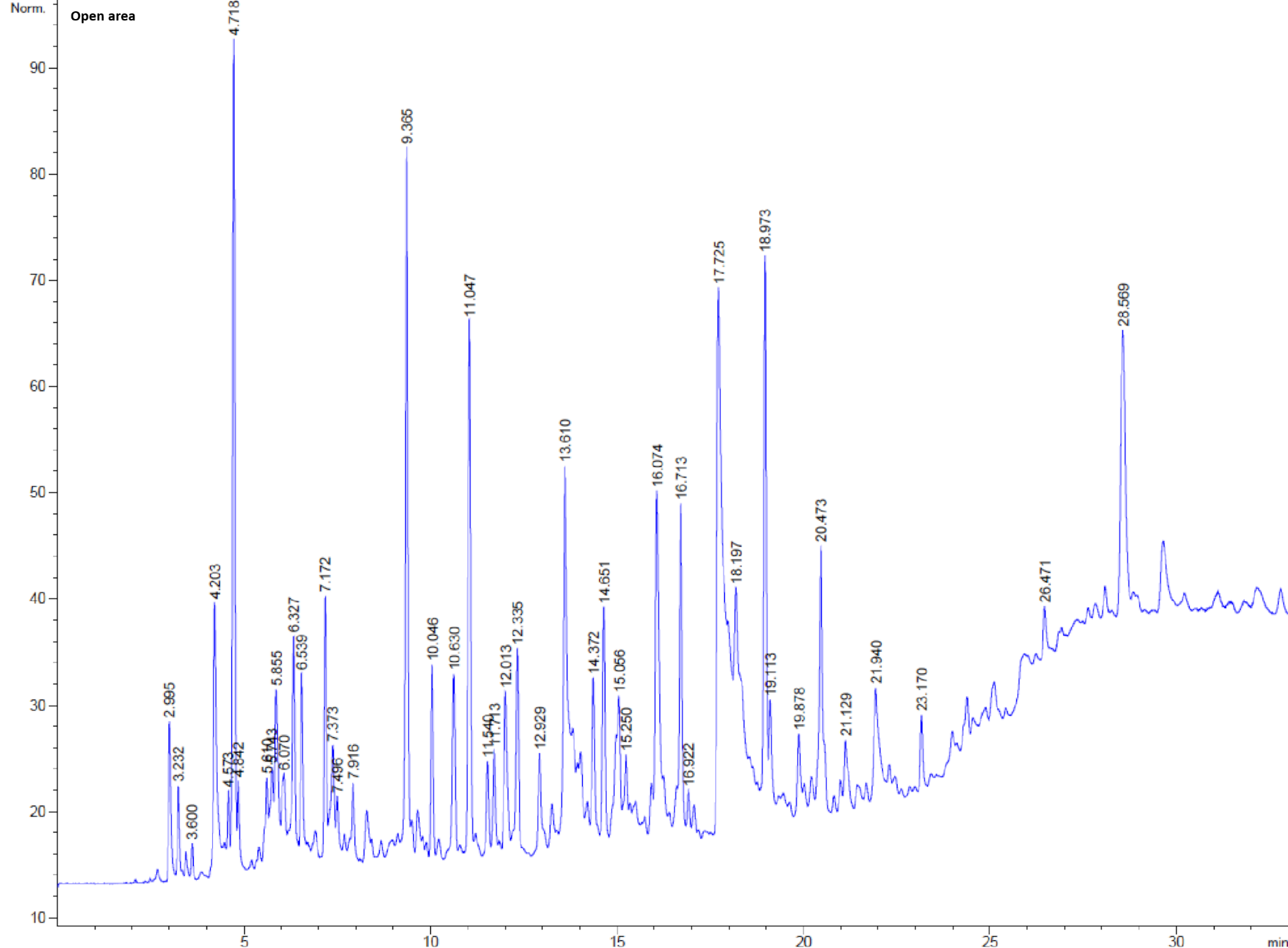


**Supplementary Figure 1**. Chromatogram obtained during the analysis of analyte samples collected from the air over the open area.


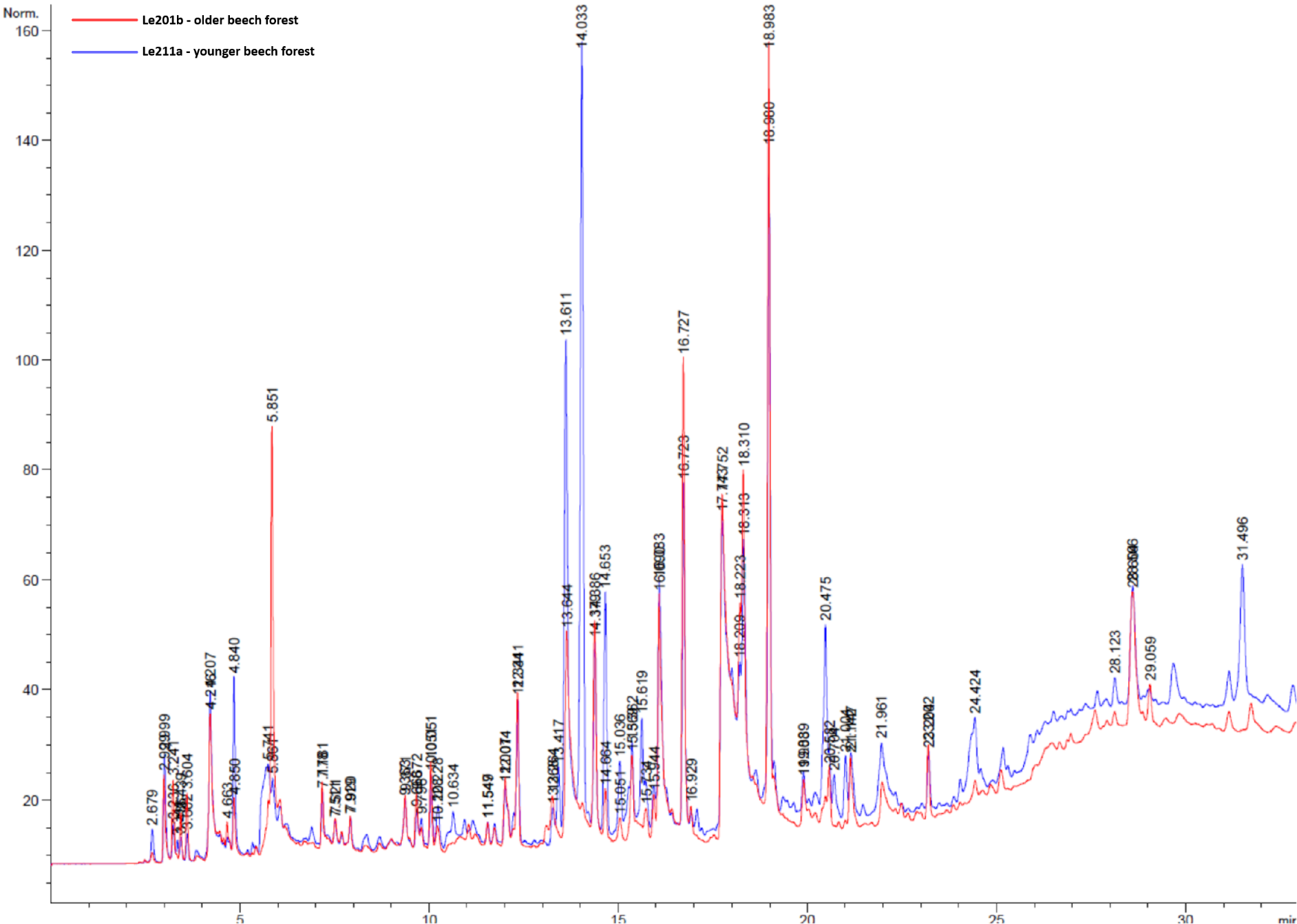


**Supplementary Figure 2**. Chromatogram obtained during the analysis of analyte samples collected from the air over the older and younger beech forest area.


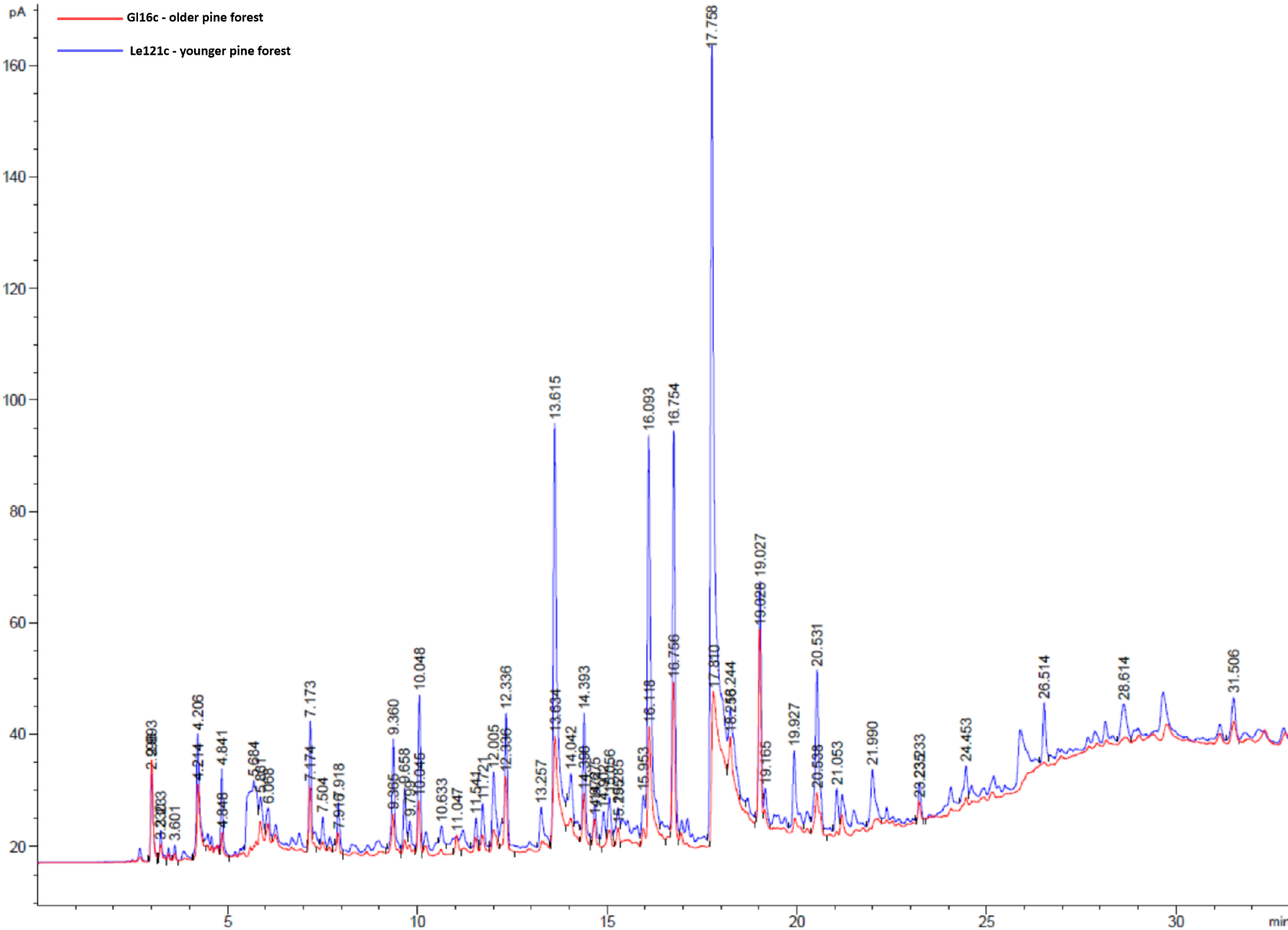


**Supplementary Figure 3**. Chromatogram obtained during the analysis of analyte samples collected from the air over the older and younger pine forest area.

**Supplementary Table 1**. Information about the retention times of the representatives of terpenes determined in the investigated air over the forest and open area.

| Retention time | Compound |
| --- | --- |
| 13.630 | α-pinene |
| 14.071 | camphene |
| 14.696 | β-pinene |
| 15.409 | 3-carene and α-terpinene |
| 15.675 | D-limonene |
